# New energy with ZnS: novel applications for a standard transparent compound

**DOI:** 10.1038/s41598-017-17156-w

**Published:** 2017-12-01

**Authors:** Pino D’Amico, Arrigo Calzolari, Alice Ruini, Alessandra Catellani

**Affiliations:** 1 Dipartimento di Scienze Fisiche, Informatiche e Matematiche, Università di Modena e Reggio Emilia, I-41125 Modena, Italy; 2 Istituto Nanoscienze CNR-NANO-S3, I-41125 Modena, Italy

## Abstract

We revise the electronic and optical properties of ZnS on the basis of first principles simulations, in view of novel routes for optoelectronic and photonic devices, such as transparent conductors and plasmonic applications. In particular, we consider doping effects, as induced by Al and Cu. It is shown that doping ZnS with Al imparts a n-character and allows for a plasmonic activity in the mid-IR that can be exploited for IR metamaterials, while Cu doping induces a spin dependent p-type character to the ZnS host, opening the way to the engineering of transparent p-n junctions, p-type transparent conductive materials and spintronic applications. The possibility of promoting the wurtzite lattice, presenting a different symmetry with respect to the most stable and common zincblende structure, is explored. Homo- and heterojunctions to twin ZnO are discussed as a possible route to transparent metamaterial devices for communications and energy.

## Introduction

Research into the exploitation of the peculiar optical properties of zinc sulfide started several years ago. Zinc sulfide is one of the most important II-VI compound semiconductors: it has a large (3.6 eV) direct band gap, a sizable exciton binding energy of 39 meV at room temperature^1^, and it may in principle support both n- and p-doping. Moreover, ZnS is a low-cost, environmentally benign compound, with convenient mechanical properties, such as good fracture strength and hardness. ZnS exhibits polymorphism since two main crystalline forms can be observed, namely the most stable (below 1290 K) zincblende (ZB) and the high-temperature and synthetically feasible^2^ allotrope with wurtzite (WZ) symmetry. ZnS can be transparent in an extremely wide energy range, with a very large transmittance from visible wavelengths to just over 12 micrometers. Indeed, among the many proposed ZnS-based device applications, one can find solar cells, liquid crystal (flat panel) displays, light-emitting diodes and sensors^1,3–5^ transmission windows for visible and infrared optics, due to its optimal performances as optical material. Furthermore, various ZnS-based nanostructures have been successfully synthesized, including nanowires^1,3^, nanoribbons^4^ and nanotubes^5^, that may be easily integrated in nanoscale devices. Among these, particular attention has been payed to nanostructures and multilayers composed of ZnS and its companion ZnO^6^, that find relevant applications in piezotronics^7^, photovoltaics^8–10^ and photodetectors^11^. The combination of ZnS with other materials, such as ZnO, is also strategic in view of novel imaging and sensing applications, e.g. in the field of plasmonics - the main effects being the huge field enhancement and strong localization at the interface - or in the design of hyperbolic metaterials^12^, where one exploits the indefinite (hyperbolic) dispersion of the refracted electromagnetic wave.

Despite the huge amount of data and applications, the electrical and optical response of ZnS in the far-IR and THz range remain poorly explored, albeit this is an extremely relevant spectral region for e.g. biosensing and high frequency electronics. The excitation of plasmons in the THz range for example could be exploited for the rectification of THz radiation in field-effect transitor (FET) devices, thus pushing the realm of todays electronics from GHz to THz, realizing more compact sources and reaching the speed of photonic devices. Furthermore, the possibility to combine and build with other materials effective metamaterials with optical properties that can not be found in natural compounds, would allow to tailor reflection/transmission of optoelectronic waves in solid systems, opening the way to ambitious applications, such as realization of hyperlenses, waveguides and subwave diffractive systems.

In this paper, we present a first-principles investigation of the electronic, optical and plasmonic properties of doped ZnS, by focusing on its performance as a transparent and conducting material in the THz and far/mid-IR. In particular, we provide a microscopic study of doping with either substitutional Al or Cu, both at Zn sites: confirming preliminary results^13^, we show that doping ZnS with Al imparts a n-character, while Cu doping induces a spin dependent p-type character to the ZnS host. To realize the full functionality and capabilities of semiconductor electronics, both electron (n) and hole (p) type conductivities are required, although useful unipolar devices can be also devised. Simultaneous transparency and conductivity are unusual in p-type as compared to n-type semiconductors: only a few wide-band-gap inorganic materials have been demonstrated to exhibit the necessary electronic and structural features for realization of effective p-type doping and most of them include rare and expensive elements such as lantanides or heavy metals as In or Pb (see e.g. refs^14–16^). Our results thus strongly support the possibility to design non pollutant transparent p-n homojunctions and novel p-type transparent conductive (TC) materials, with spin resolved transport conduction.

We furthermore investigate the effect of doping on the possible plasmonic activity of realistic (ZnS) bulk and interfacial (ZnO/ZnS-based) systems, with an eye to the realization of novel, clean and safe metamaterials for communication, energy and (bio-)sensing applications.

## Results and Discussion

### Transparent conductors

**Figure 1:**
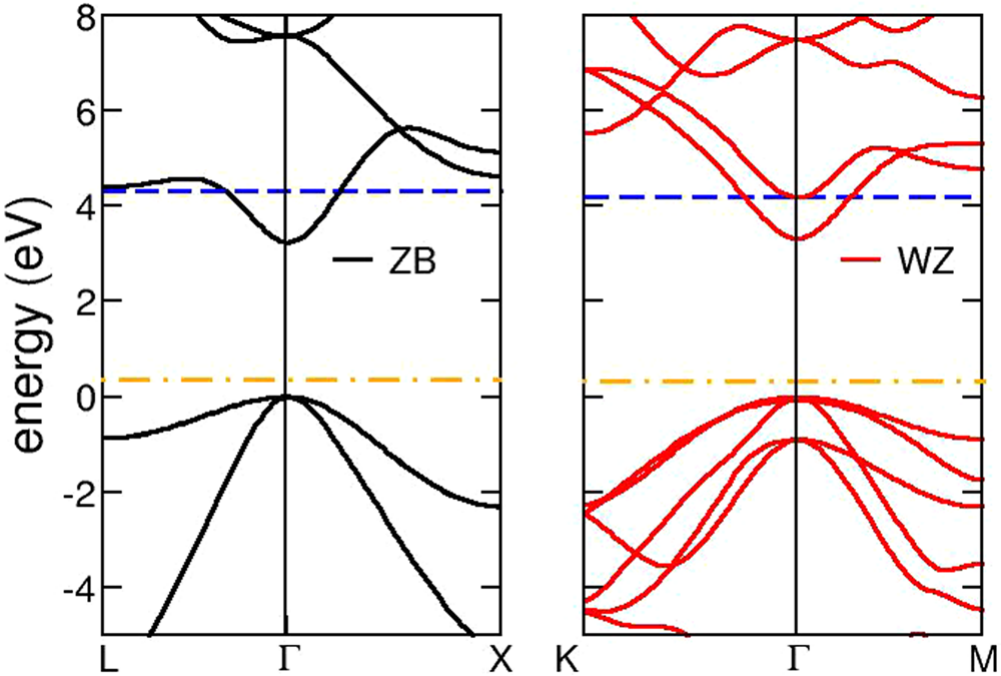
Band structure of ZnS for the two different crystal structures, zincblende (left-panel) and wurtzite (right-panel). Blue (orange) dotted lines represent the Fermi level for Al-doped (Cu-doped) ZnS. The valence band maximum of clean ZnS is used as energy scale reference (0 eV) for both lattices.

The ZnS electronic band structure is shown in Fig. 1 for both possible tetrahedral symmetries. The DFT + U approach (see Method) leads to a band gap of ≃3.25 eV for both the zincblende (Fig. 1-left panel) and the wurtzite (Fig. 1-right panel) lattices, in good agreement with the experimental 3.6 eV value. The conduction band in ZB symmetry is characterized by a single band along the full Brillouin zone with an almost parabolic shape around the Γ point (see left panel, Fig. 1), which implies a quasi-free electron gas behavior for conduction electrons upon doping. The effective masses for electrons *m* ^*^, calculated around Γ from band structure along the *L* − Γ − *X* line, is 0.24 *m*_0_, in agreement with the reported experimental value (0.28 *m*_0_)^17,18^. The two corresponding hole effective masses are instead 0.86 *m*_0_ and 0.18 *m*_0_.

For the less stable WZ structure the lowest conduction band, although still parabolic, is less isolated than in the ZB case: indeed, the energy difference between the conduction band minimum at Γ and the upper band is less than 1 eV, while in ZB the two lowest conduction bands at Γ are separated by ≃4.3 eV, see Fig. 1). This implies that after filling of the lowest conduction band upon doping, the minimum energy difference between occupied and empty states (i.e. interband transitions) lies in the IR range, reducing the intrinsic transparency range. Furthermore, the electron effective mass around Γ is ${m}_{e}^{\ast }=0.30\,{m}_{0}$ for the WZ lattice, i. e. larger than for the ZB case: these two features point to possibly worse transport and optical efficiency of the WZ lattice upon n-doping.

Furthermore, at variance with ZB, in the absence of doping, the WZ lattice sustains a macroscopic polarization along the stacking direction (*z*), which for ZnS is *P*_*z*_ = 5×10^−3^ *C*/*m* ^2^, as calculated via the Berry phase approach included in the Quantum ESPRESSO package^19^. This small value, still comparable to those predicted for the wurtzite structure of similar compounds, e.g. ZnO^20^, SiC^21^ and BeO^22^, is linked to the reduced stability of the hexagonal vs zincblende structures, and to the fact that significant differences between the two lattices appear only at third neighbors. Nonetheless, the presence of a spontaneous polarization may induce relevant effects in the first stages of strongly non-adiabatic growth processes, such as favoring one lattice over the other, especially for thin films and nanostructures^6^.

Al/Cu doping effects are accounted for, with the same degree of accuracy, by considering that these dopants typically assume Zn-substitutional sites within the experimental solubility limit (≃10%), as it is the case for Al:ZnO^23,24^. In this doping range, the crystalline order of the ZnS host is only slightly perturbed, as also suggested by experimental findings^13,25,26^.

#### Al:ZnS

As a substitutional defect, (i) Al does not induce the formation of defect states in the ZnS band gap, as also evident from the density of states (DOS, not shown), and (ii) Al donates its 3*p* electron to the ZnS conduction band, without remarkably changing the shape and the curvature of the conduction band minimum (upon doping, the calculated effective mass of the e.g. ZB phase is ${m}_{e}^{\ast }=0.22\,{m}_{0}$ with respect to ${m}_{e}^{\ast }=0.24\,{m}_{0}$ for the pure system). In virtue of the peculiar parabolic behavior at the ZnS conduction band bottom, Al-doping induces the formation of a free electron gas in the host system: the blue lines in the electronic band structures shown in Fig. 1 indicate the Fermi energy position in the Al-doped WZ and ZB systems. From inspection of the electronic band structures for both symmetries, and the calculated effective masses, it is clear that the zincblende phase of ZnS is better suited as a transparent conducting material upon doping, while the small energy difference between the lowest conduction bands in the wurtzite symmetry is expected to affect the transparency properties of the system, favoring IR interband transitions, as discussed above.

The formation of a conducting electron gas is the typical fingerprint of an intrinsic n-type conductor and the fundamental prerequisite for a plasmonic material. Some suggestions of plasmonic activity in ZnS-based systems have indeed been provided, especially for the case of composite materials (metamaterials) or core-shell plasmonic nanoparticles^27,28^, where a tunable localized surface plasmon resonance can be exploited^29^.

**Figure 2:**
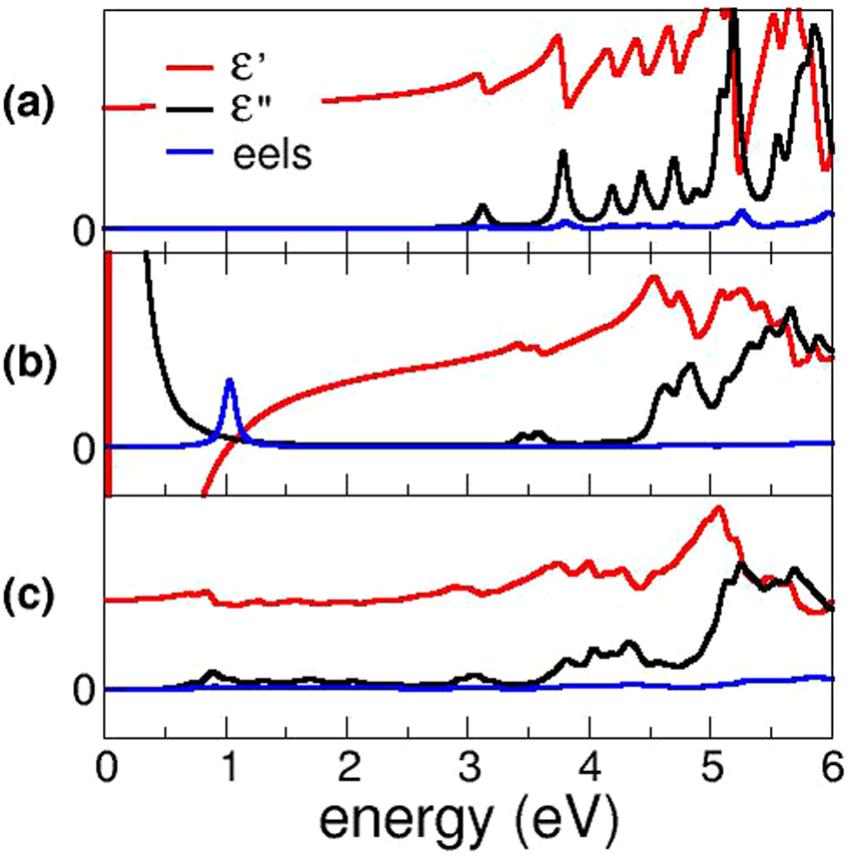
Imaginary (black) and real (red) part of the dielectric function and eels function (blue) for the systems studied in this work. From top to bottom: (**a**) clean ZnS, (**b**) Al:ZnS (Al content ≃1.6%) and (**c**) Cu:ZnS (Cu content ≃3.2%). Only the results for the zincblende host lattice are shown, as the wurtzite case presents only minor modifications.

The real and imaginary part of the dielectric function for Al:ZnS in both wurtzite and zincblende structure were calculated by using the Drude-Lorentz model^30^ (see Method). Figure 2 shows a comparison of the response functions for clean (panel a) and Al-doped (panel b) ZnS in the zincblende structure. In both cases, the first peak in the imaginary part of the dielectric function is in the UV range, corresponding to the interband valence-to-conduction absorption edge, while the transparency is preserved in the visible range, in agreement with experiments^31^. At lower frequencies the optical behavior closely resembles the one of an ideal Drude metal: for *ω* → 0^+^, both *ε*′ and *ε*″ display a divergence to −∞ and + −∞ (the damping factor being related to scattering processes associated to the electrical resistivity), in agreement with the formation of a free electron gas upon Al-doping.

The loss function is also shown in Fig. 2b: its most marked spectral feature is related to the presence of a sharp peak corresponding to the low energy plasma frequency (*ω*_*p*_) of the system, that amounts to 1.02 eV for zincblende and to 0.83 eV for wurtzite Al:ZnS (not shown), in the energy region where *ε*′ = 0 and *ε*″ ≪ 1. As explicitly demonstrated for the similar case of Al-doped ZnO^32^, *ω*_*p*_ is not an intrinsic property of the material, as it happens for noble metals, but can be properly engineered by controlling the electron density, i.e. by changing the doping and defect concentration^33^. The tunability of the plasma frequency has important implications from the application viewpoint, since it allows to engineer the operating frequency region of the material in connection with other dielectric layers for realization of metamaterials and surface plasmon polariton (SPP) waveguides.

The bulk plasma frequency in the dipole approximation is linked to the free electron charge density *N*_*e*_ available in the conduction band of a doped compound via

1 $${\omega }_{p}=\sqrt{\frac{4\pi {e}^{2}{N}_{e}}{m}},$$

where *m* = m*m_0_ is the effective mass for electrons, and *m*_0_ is the electron rest mass. For the zincblende (wurtzite) structure we find *N*_*e*_ = 1.3 × 10^20^ cm^−3^ (*N*_*e*_ = 1.1 × 10^20^ cm^−3^). Correspondingly, the calculated (Boltzmann) electron conductivity for ZB is *σ*_*el*_/*τ* = 1.68 × 10^21^ S s^−1^, ^34^ where *τ* is the relaxation time. By considering that larger Al dosages would actually correspond to a larger amount of free charge injected into the ZnS conduction band, and that 10^19^ cm^−3^ is considered as the practical minimum threshold for sustaining propagating surface plasmon polaritons in extended structures, our results indicate that doping values higher than 1.6% are required to actually generate plasmonic excitations in Al:ZnS. Higher dopant contents are still achievable without alloying, a result compatible with the experimental doping rates, ranging up to 10% Al concentration, with optimal dosages of ≈6% Al content^35^. Higher charge densities can be also obtained in nanostructures or layered systems, where confinement effects can severely affect this picture. Some interface effects are discussed below.

#### Cu:ZnS

The effects of Cu_*Zn*_ substitutional doping are also presented, with the aim to investigate a potential p-type counterpart of Al:ZnS, that was demonstrated to behave as a n-type transparent conductor. Indeed, copper is a favored choice to obtain p-type doping of several compounds, including in particular copper delafossites^14,36^, which are at present among the few p-type TCs that reach the device standard, although still with much lower performances than the most common n-type TCs.

**Figure 3:**
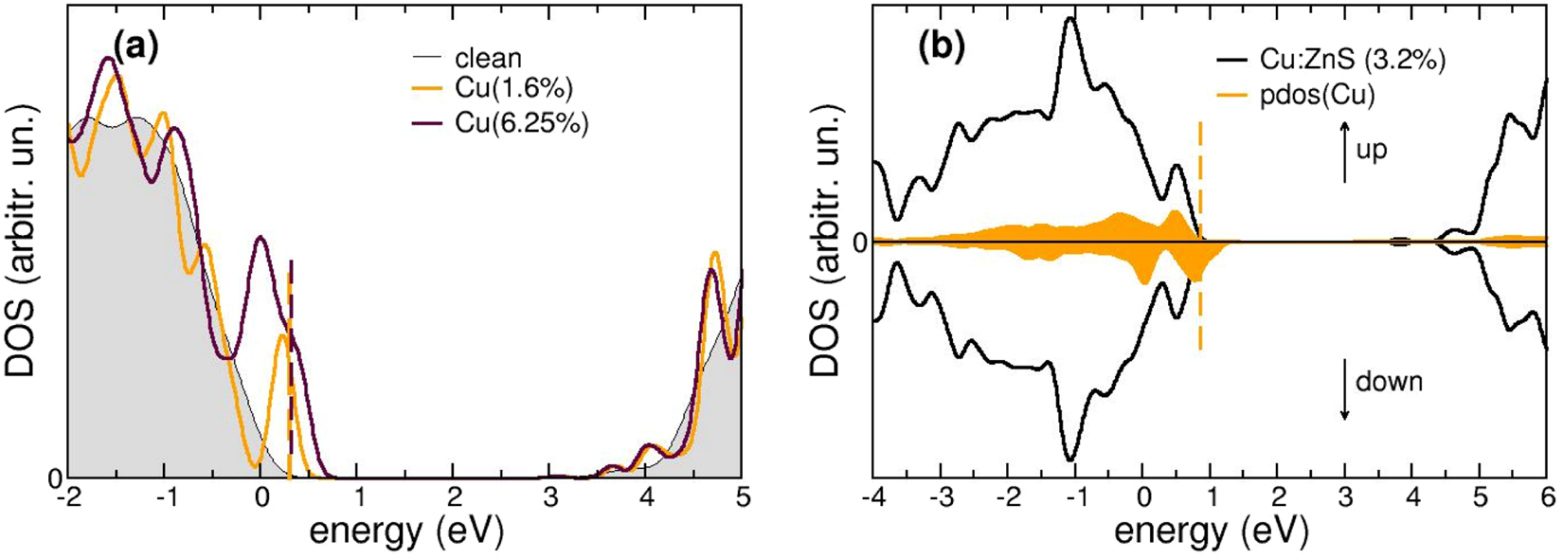
(**a**) Modification of the total DOS around the band gap region of ZB-ZnS upon Cu doping at different dosages. (**b**) Spin-resolved DOS of Cu-doped (3.2%) ZB-ZnS; spin-down states are reported with negative values. The projected DOS on Cu-derived states is also indicated in orange. In all panels, the Fermi Level of the undoped system is chosen as reference (0 eV) for the energy scale, while the dotted line represents the Fermi level of the doped structure.

Being interested in TCs, we did not consider the effect of complex formation with other defects, such as S vacancies, Cu clustering or amorphous samples, which might influence Cu solubility^37^, but we sticked at the study of Cu doping in substitutional Zn sites. The optimized structure does not show modifications beyond first neighbors (bond distances shortened of ≃0.07 Å for maximum dosage), at least at a dopant concentration up to the solubility limit (≃1.6–10%, 1–6 Cu atoms in a 64 atoms cell), in agreement with experiments^13,38^, as previously anticipated. In this case however, the host band-structure is modified in the close proximity of the valence band maximum (VBM), as semi-occupied defect states appear in the pristine ZnS band-gap close to VBM (see Fig. 3) that shift the Fermi level, which is moved in the valence band of the defective sample (≃0.35 eV above VBM in the pristine host, orange line in Fig. 1). These Cu-S derived states further foster s-d hybridization: this increases the dispersion of the outermost valence bands and induces a beneficial effect on transport properties, via a reduction of hole effective masses. The proper choice of the p-dopant content is thus the result of a balance between this modification of the valence band top states and possible structural deformations, linked to higher dosages. The ZnS hole conductivity at 3.2% doping is *σ*_*h*_/*τ* = 1.23×10^20^ S s^−1^ ^34^. In the hypothesis that both carriers have similar relaxation times *τ*, this leads to *σ*_*el*_/*σ*_*h*_ = 14 which is in the range of reported device-quality experimental data (see ref.^13^. and e.g. www.azom.com). More importantly, Cu:ZnS presents a net total magnetization of 1.00 Bohr mag/Cu atom/unit cell, as revealed also in the projected density of states shown in Fig. 3(b). At variance with Al, which is a degenerate dopant, it must be noted that the observed p-conductivity derives from the only partial saturation by copper of the charge depletion associated to the substituted Zn atom. This is reflected by the Fermi level pinning at defect states, as revealed by the comparison of DOS for different Cu contents reported in Fig. 3. Similarly, the net magnetization is associated to unpairing of neighboring S-orbitals induced by the partially saturated Zn vacancies, as it happens for the case of ZnO^39–41^.

These results are particularly exciting in view of potential applications in the field of transparent spintronics, as well as for tunable p-n transparent homojunctions and devices, which can find application in e.g. photocatalysis under visible-light irradiation, (bio-)sensing etc. On the other hand, no plasmonic activity is predicted for this p-type TC: indeed, the response function *ε* displays only small modifications with respect to the one calculated for the unperturbed host in the frequency range of interest - i. e. below the optical transitions - as shown in the lowest panel of Fig. 2.

Similar considerations hold for the wurtzite phase. Here the distribution of bands in the pristine undefective host promotes p-doping at lower Cu dosages (see Fig. 1).

### Surface-plasmon polariton

The potential of ZnS-related systems for plasmonic applications includes the possibility to exploit ZnS-based metal/dielectric interfaces that can sustain surface-plasmon polariton formation and propagation. A SPP is a transverse electromagnetic excitation that propagates at a planar conductor/dielectric interface: it originates from the coupling between the electromagnetic field of light and the resonant plasma oscillation of the conduction electrons of the metal. The huge field enhancement and the strong localization at the interface can lead to potentially interesting applications across many fields including spectroscopy, nanophotonics and biosensing.

A convenient way to describe SPPs is the dispersion relation that correlates the frequency of impinging light *ω* with the wave vector *k*_||_ in the direction of propagation of SPP. The general equation that defines this relation is

2 $${k}_{||}=\frac{\omega }{c}Re[\sqrt{\frac{{\tilde{\varepsilon }}_{m}{\tilde{\varepsilon }}_{d}}{{\tilde{\varepsilon }}_{m}+{\tilde{\varepsilon }}_{d}}}],$$

where ${\tilde{\varepsilon }}_{m}$ and ${\tilde{\varepsilon }}_{d}$ are the complex dielectric functions of the metal and the dielectric layers, respectively, and c is the speed of light in vacuum.

**Figure 4:**
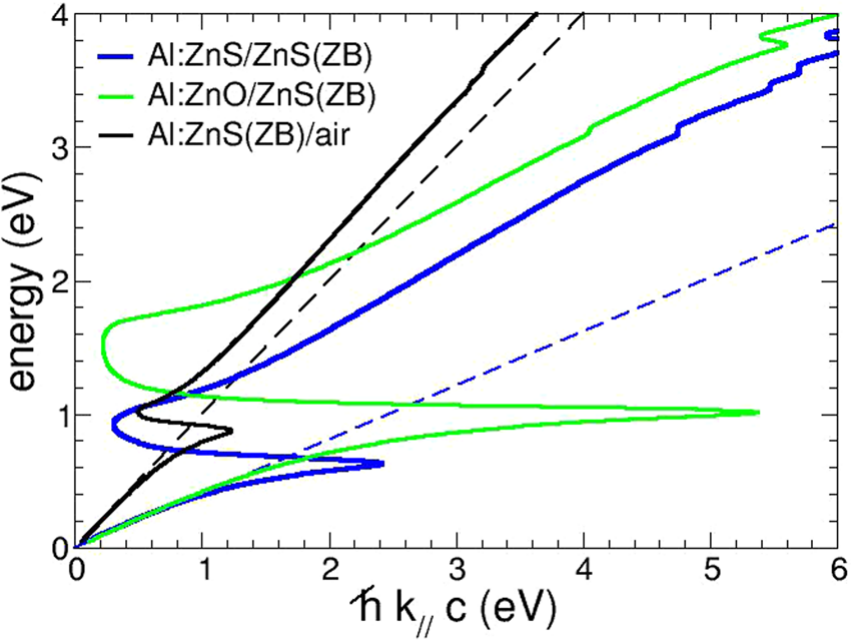
SPP dispersion relation for ZnS-based interfaces. Dashed lines are the dispersion relations for light in the corresponding dielectrics.

**Figure 5:**
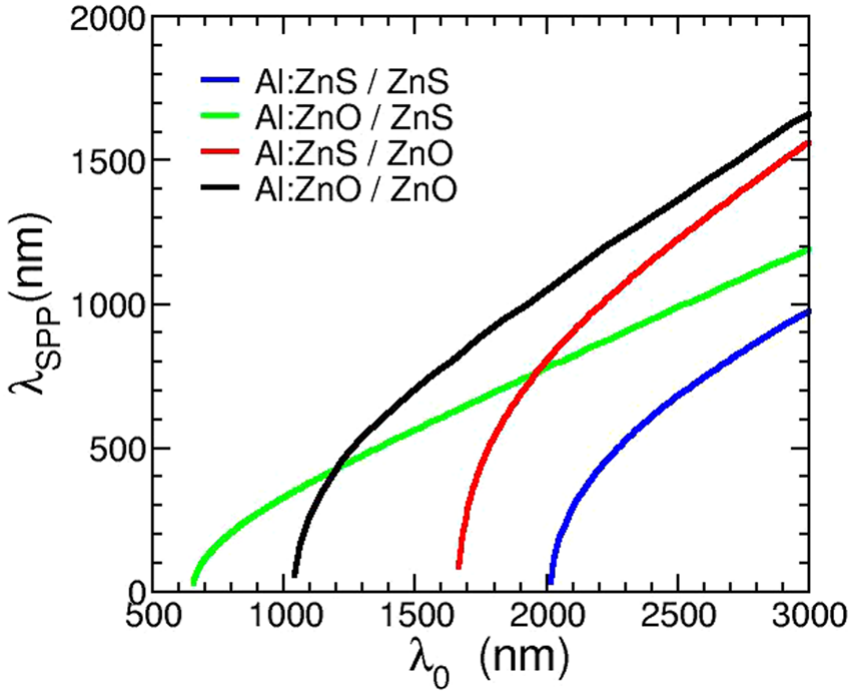
SPP wave-length calculated for various combination of metal/dielectric interfaces. The doping percentage of Al:ZnO and Al:ZnS is ≈1.6%.

By assuming ZnS as the dielectric component and Al:ZnS (at 1.6% Al content) as the metallic one, we can obtain the dispersion relations for the Al:ZnS/ZnS interface. Figure 4 shows such dispersion relation for the Al:ZnS/ZnS interface, in comparison with Al:ZnS/air and Al:ZnO/ZnS (Al = 1.6%). The light line of the corresponding dielectrics is also indicated as a dashed line. We first highlight the common features of all these interfaces. At low energies, the SPP lays close to the corresponding light line, as in the ideal case; the fact that *k*_||_ is always larger than the corresponding light line is indicative of the non-radiative nature of the SPP modes, that are propagating modes bound at the interface in this energy region. Close to the resonance condition (*ω* = *ω*_*SPP*_, or *ε*_*m*_ = −*ε*_*d*_) they bend back, cross the light line and become dispersive quasi-bound, although not purely imaginary, modes. The SPP dispersion relations are quite similar for both ZnS-based interfaces, namely WZ and ZB, and the curves for different lattices almost superimpose; Al:ZnS surfaces (namely the interface with air) can hardly sustain a SPP mode (black line. Fig. 4). Remarkably, the interface between ZnS and Al:ZnO provides enhanced results in terms of wave vector and transition energies, as shown in Fig. 4. The ZnO/ZnS heterojunction is therefore quite promising, and can be optimized also in view of SPP applications in the IR range. In Fig. 5, we show in fact the SPP wavelength for mixed dielectric/metal interfaces based on ZnS and ZnO materials, that we compare with homogeneous Al:ZnS/ZnS and Al:ZnO/ZnO^32^ interfaces (all systems were here considered in their most stable crystalline structure, i.e. ZB for ZnS and WZ for ZnO). The SPP wavelength defines the period of a SPP oscillation: our results show that different conductor/dielectric configurations correspond to different SPP periodicity, namely Al:ZnS induces lower (almost one half) SPP wavelengths than the corresponding Al:ZnO-based interfaces; furthermore the larger slope of the curves for ZnO with respect to ZnS as a dielectric points to a larger transported energy for the propagating SPP wave. A proper choice of the material thus allows a tuning of the SPP properties in terms of both activation and intrinsic energy of the SPP mode which define the dimensions of the final optical devices (e.g. Bragg scatterers).

### Hyperbolic metamaterials

Up to now we considered the optoelectronic properties of doped ZnS in view of (traveling plasmons) SPP applications, and we demonstrated that one can indeed obtain collective excitations with low plasma frequency *ω*_*p*_ to be exploited in the IR range. In order to pursue further plasmonic applications in the low energy range (down to far/mid IR), we have investigated the optical response of a metal-insulator bulk interface composed by Al:ZnO/ZnS in search for an all-dielectric hyperbolic metamaterial for optic/photonic applications. In this field, indeed, the possibility to tailor the optical response, rather then the presence of large contents of free charges, is the fundamental requirement^42^.

Metamaterials are engineered assemblies of different materials with designed physical properties such as permittivity and permeability that vary within dimensions much smaller than the wavelength of the incident waves. They find applications in a number of novel intriguing photonic applications such as imaging, cloaking, sensing and waveguiding^43^. Among these, a special novel class is covered by hyperbolic metamaterials (HMM)^12^, their main property being an indefinite, therefore hyperbolic, dispersion of the refracted electromagnetic wave; this behavior originates from the fact that one principal component of their permittivity or permeability effective tensors has an opposite sign with respect to the other two principal components.

If one considers these artificial media as effective materials, it is possible and intuitive to use relations of standard bulk optics for the description of their response function in the long-wavelength limit, when the variation of the electromagnetic field over one periodic layer of the structure is small. For transverse magnetic waves impinging on a two-layers system, as the one that we study here, the effective dielectric function can be expressed^12^ by:

3 $${\varepsilon }_{eff}={\varepsilon }_{||}+{\varepsilon }_{\perp }$$

where the $\varepsilon$ components parallel and perpendicular to the optic axis (namely the components along the directions perpendicular or parallel to the interface in a multilayer stacked system) are defined as

4 $${\varepsilon }_{||}^{-1}=(\frac{{d}_{m}/{\varepsilon }_{m}+{d}_{d}/{\varepsilon }_{d}}{{d}_{m}+{d}_{d}}),\,{\varepsilon }_{\perp }=(\frac{{d}_{m}{\varepsilon }_{m}+{d}_{d}{\varepsilon }_{d}}{{d}_{m}+{d}_{d}})$$

where *d*_*m*_ (*d*_*d*_) and *ε*_*m*_ (*ε*_*d*_) are the thickness and the dielectric function of the metal (dielectric). In this case, it is possible to obtain negative refraction when *ε*_*||*_ *ε*_⊥_ < 0.

**Figure 6:**
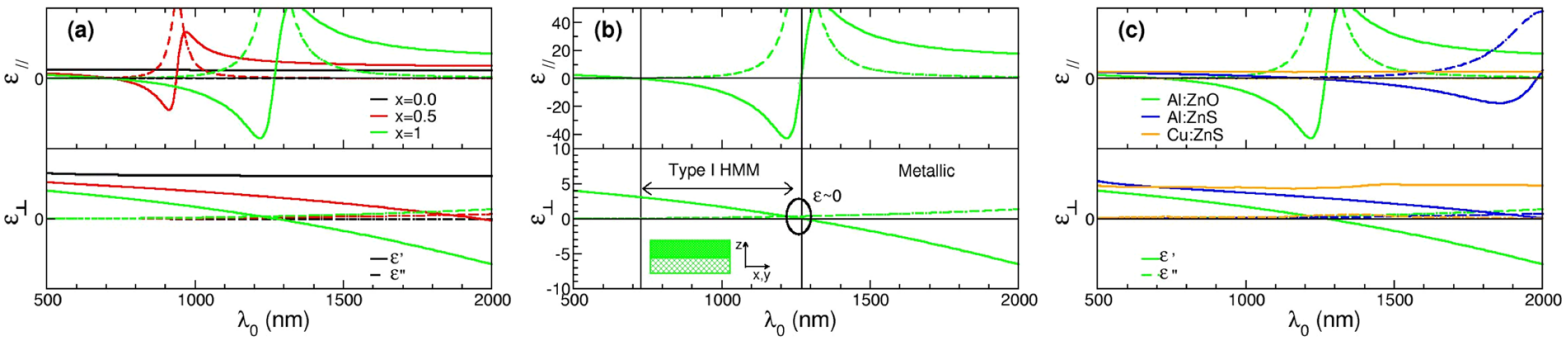
Effective medium dielectric function for metal/dielectric interfaces composed by Al:ZnO/ZB-ZnS at different thickness ratios *x* = *d*_*m*_/*d*_*d*_ between the two constituents (panel a), and for different doped dielectrics interfaced to ZB-ZnS at *x* = *d*_*m*_/*d*_*d*_ = 1 (panel c); in (**a**) the curves for pure ZnS (x = 0) are included for comparison. Panel b highlights the different regions of the spectrum, and the corresponding average behavior of an Al:ZnO/ZB-ZnS bilayer with equal thicknesses (*x* = 1); a sketch of the structure is depicted in the lowest right part of the panel. The strong oscillations in *ε*_||_ correspond to the onset of the surface plasmon polariton of the metallic layer and show marked dependence on the *d*_*m*_/*d*_*d*_ ratio.

In Fig. 6 we plot the effective medium dielectric function for different interfaces where ZnS always plays the role of the dielectric, while the metal layer is doped ZnS or Al:ZnO (results are presented for the ZB symmetry - WZ being extremely close and not distinguishable on the scale of the plots). As detailed in Fig. 6b, different ranges emerge where the metal-dielectric layered structure presents metallic, espilon-near-zero (ENZ) or hyperbolic properties. In particular for a layered structure with equal thicknesses of the constituents (*x* = *d*_*m*_/*d*_*d*_ = 1), the HMM regime appears for 725 < *λ* < 1250 nm, while the transitional ENZ regime, where *ε*_*||*_ ≥ 0 and *ε*_⊥_ ≃ 0, occurs around *λ* ≃ 1250 nm. Similarly to what already demonstrated for the Al:ZnO/ZnO interface^44^, our results show that layered systems composed by Al:ZnO/ZnS or Al:ZnS/ZnS behave as metamaterials with negligible transparency in the mid-far IR range; furthermore, two parameters emerge for tuning the operation range, namely the dopant content, to optimize the plasma frequency via donor charge density, and the thickness ratio of the constituents.

On the other hand, Cu-doping is not fruitful for this kind of applications, as the sign of *ε*_*||*_ *ε*_⊥_ is always positive, in the full range (Fig. 6c).

## Conclusions

We presented a first principles investigation of the electrical and optical properties of doped ZnS. Our results highlight plasmonic activity for Al:ZnS and indicate that this system acts as a transparent and conducting material, coupling transparency in the visible/infrared range and metal-like conductivity in the far/mid-IR for experimentally feasible doping concentrations. The intrinsic electronic properties of the ZnS host facilitate the formation of a free electron gas upon n-doping, which can be excited in the near/mid-IR range to give plasma oscillations. In the case of Cu-doped ZnS, we confirm the experimental observation of p-type doping and reduced transparency. In this specific case, we however remark that our results show that p-type conductivity originates from the uncomplete compensation of charge depletion induced by the Zn vacancy: in fact, Cu-doped ZnS does not behave like a Drude metal, as evidenced by the lack of plasmonic signatures.

The overall reported optical properties of metal-doped ZnS derivatives in the mid-IR regime support their application for the realization of hybrid architectures between dielectric materials, for the development of p-type TCs, bipolar homojunctions, and all-dielectric metamaterials, where the combination of lattice matched interfaces, non pollutant, biocompatible constituents pave the way to the design of novel transparent devices for communication and (bio)-sensing.

## Methods

The electronic properties of pure and doped ZnS were calculated within the density functional theory (DFT) framework, as coded in the Quantum ESPRESSO package^19^. We adopted the Perdew-Burke-Ernzerhof generalized gradient approximation (PBE-GGA)^45^ for the exchange-correlation functional, ultrasoft pseudopotentials^46^ for all the atomic species, a plane wave expansion of wavefunctions (charge) up to a cutoff energy of 30 (240) Ry. The *3d* electrons of Zn have been explicitly included. A Hubbard-like potential with U = 13.7 and 3.51 eV^47,48^ is applied on the *d* and *p* orbitals of zinc and sulfur, respectively^49–52^. This is an efficient and computationally inexpensive way to correct for the severe underestimation of the band gap and the wrong energy position of the *d*-bands of the Zn atoms^47,48,51,52^. On the other hand U values for the dopant species, namely Al and Cu, as immersed in the host matrix, are negligible^53^. The adopted U values for both elements have been calculated using the pseudo-hybrid implementation of DFT + U (ACBN0)^47,48^. A detailed description of the method and tests on similar systems close to the one studied here can be found in literature^20,47,48,52,54^.

All the studied systems (i. e. the ZnS, Al:ZnS, Cu:ZnS bulk structures) were investigated in both their possible tetrahedral symmetries, namely zincblende and wurtzite, by using periodically repeated orthorombic supercells, with 64 atoms: Thus e.g. the 1.6% Al doping regime was simulated by including one substitutional Al atom at a Zn site. We used a 4 × 4 × 4 k-point grid to sample the complete Brillouin zone of the system. All structures were relaxed until forces on all atoms became lower than 10^−3^ eV/A.

The optical properties of the bulk-like systems were obtained by calculating the dielectric function by means of a generalized Drude-Lorentz expression of the macroscopic complex dielectric function $\hat{\varepsilon }(\omega )$ ^30^, as implemented in the code *epsilon.x*, also included in the Quantum ESPRESSO suite. The optical properties were calculated with an increased k-point sampling, up to a 8 × 8 × 8 mesh. Norm-conserving pseudopotentials and cutoff energy of 80 Ry (120 Ry) were employed fo Al (Cu) doped systems. Here, both intra-band (Drude-like) and inter-band (Lorentz-like) contributions are explicitly taken into account. Once the complete dielectric function is known, the electron energy loss function can be easily obtained as $L(\omega )=Im\{-\mathrm{1/}\hat{\varepsilon }\}$.

### Data availability

The datasets generated during and/or analysed during the current study are available from the corresponding author on reasonable request.
